# Semi-in vitro detection of Mg^2+^-dependent DNase that specifically digest mitochondrial nucleoids in the zygote of *Physarum polycephalum*

**DOI:** 10.1038/s41598-022-06920-2

**Published:** 2022-02-22

**Authors:** Naoki Urakawa, Satoru Nakamura, Mariko Kishimoto, Yohsuke Moriyama, Shigeyuki Kawano, Tetsuya Higashiyama, Narie Sasaki

**Affiliations:** 1 Division of Biological Science, Graduate School of Science, Nagoya University, Furo-cho, Chikusa-ku, Nagoya, Aichi 464-8602 Japan; 2 Center for the Development of New Model Organisms, National Institute for Basic Biology (NIBB), 38 Nishigonaka, Myodaiji, Okazaki, Aichi 444-8585 Japan; 3 Science and Technology Group, Okinawa Institute of Science and Technology Graduate University (OIST), 1919-1 Tancha, Onna-son, Okinawa, 904-0495 Japan; 4 Functional Biotechnology PJ, Future Center Initiative, The University of Tokyo, 178-4-4 Wakasiba, Kashiwa, Chiba 277-0871 Japan; 5 Institute of Transformative Bio-Molecules (WPI-ITbM), Nagoya University, Furo-cho, Chikusa-ku, Nagoya, Aichi 464-8601 Japan; 6 Department of Biological Sciences, Graduate School of Science, The University of Tokyo, 7-3-1 Hongo, Bunkyo-ku, Tokyo, 113-0033 Japan; 7 Institute for Human Life Innovation, Ochanomizu University, 2‑1‑1 Otsuka, Bunkyo‑ku, Tokyo, 112‑8610 Japan

**Keywords:** Genetics, Molecular biology

## Abstract

The maternal/uniparental inheritance of mitochondria is controlled by the selective elimination of paternal/uniparental mitochondria and digestion of their mitochondrial DNA (mtDNA). In isogamy, the selective digestion of mtDNA in uniparental mitochondria is initiated after mating and is completed prior to the elimination of mitochondria, but the molecular mechanism of the digestion of uniparental mtDNA remains unknown. In this study, we developed a semi-in vitro assay for DNase, wherein the digestion of mitochondrial nucleoids (mt-nucleoids) was microscopically observed using isolated mitochondria from *Physarum polycephalum* and the DNase involved in uniparental inheritance was characterized. When myxamoebae of AI35 and DP246 are crossed, mtDNA and mt-nucleoid from only the DP246 parent are digested. The digestion of mt-nucleoids was observed in zygotes 3 h after plating for mating. During the digestion of mt-nucleoids, mitochondrial membrane integrity was maintained. In the semi-in vitro assay, the digestion of mt-nucleoids was only observed in the presence of Mg^2+^ at pH 7.5–9.0. Moreover, such Mg^2+^-dependent DNase activity was specifically detected in mitochondria isolated from zygotes 3 h after plating for mating. Therefore, Mg^2+^-dependent DNase is potentially involved in uniparental inheritance. Our findings provide insights into the DNase involved in uniparental inheritance and its regulatory mechanism.

## Introduction

Mitochondria possess their own DNA (mitochondrial DNA; mtDNA), which is considered a remnant of bacterial symbiosis^1^. Over the course of evolution, the size of mtDNA has shrunk markedly, and the copy number of mtDNA in individual cells has increased from hundreds to thousands. In vivo, these mtDNA are organized into mitochondrial nucleoids (mt-nucleoids), which are DNA–protein complexes that act as critical units of mtDNA inheritance. Interestingly, mtDNA is inherited from only one parent, normally maternal gametes, in almost all sexual reproductive species, including animals, plants, fungi, and protists^2–5^. This phenomenon is called maternal/uniparental inheritance.

In oogamous species, sperm cells are much smaller than egg cells. The mitochondria and mtDNA in sperm are decreased during spermatogenesis^6,7^. In general, mitochondria in sperm have a small amount of mtDNA and enter the egg upon fertilization, although it has been reported that mtDNA is completely removed during spermatogenesis in *Drosophila melanogaster*^8^. After fertilization, paternal mitochondria in embryos are selectively eliminated by the ubiquitin–proteasome system and mitophagy in monkey^9^, cow^9^, *D. melanogaster*^10^, mouse ^11^, and *Caenorhabditis elegans*^12^. In *C. elegans*, elimination of parental mitochondria by mitophagy is an important mechanism of uniparental inheritance. In contrast, in *Oryzias latipes*, the mtDNA in fertilized eggs is completely digested before the elimination of paternal mitochondria^7^.

In the gametes of isogamous species, the cell size and the amount of mtDNA and mitochondria are the same. Uniparental inheritance of mitochondria has also been reported in isogamous species^13–17^. In the true slime mold *Physarum polycephalum* and the basidiomycete fungus *Cryptococcus neoformans*, the uniparental mtDNA is selectively digested before elimination of mitochondria during zygote maturation^15,16^. Recently, in *C. neoformans*, an endonuclease G (NUC1), which has been reported to be involved in maternal inheritance in *D. melanogaster*^8^ and *C. elegans*^18^, was found to be inessential for the selective digestion of mtDNA for uniparental inheritance^16^.

Since isogamy is assumed to be the ancestral mode of sexual reproduction, elucidating the mechanism of uniparental inheritance in isogamous species is important for understanding the evolution of maternal/uniparental inheritance. However, the molecular mechanism underlying the selective digestion of uniparental mtDNA is still unknown. In this study, we focused on *P. polycephalum* to characterize the DNase involved in the digestion of mt-nucleoids for uniparental inheritance. In *P. polycephalum*, haploid myxamoebae act as isogametes. Two different mating types of myxamoebae fuse to form diploid zygotes and then develop into vegetative multinuclear plasmodium after repeated mitotic cycles without cell division^15^. *P. polycephalum* has oval shaped mitochondria and mitochondrial fusion doesn’t occur unless mitochondria have the mitochondrial fusion plasmid^19^. Each mitochondrion has one long rod-shaped mt-nucleoid, which contains 40–80 copies of the 62.9 k bp-mtDNA^20,21^. Such long mt-nucleoids help to monitor the digestion of mt-nucleoids during uniparental inheritance. After mating of myxamoebae, the long rod-shaped mt-nucleoids in approximately half of the total mitochondria become shorter and are then lost, a process that is easy to observe^15^. In addition, we have developed a method for the isolation of mitochondria from plasmodium, which contains structurally intact mt-nucleoids. Since the isolated mitochondria maintain the capacity for DNA synthesis and mtDNA synthesis occurs at specific sites in mt-nucleoids as well as in vivo^22^, it is possible that isolated mitochondria also possess DNase activity involved in uniparental inheritance.

In this study, we monitored the behavior of mt-nucleoids and mitochondria during zygote maturation of *P. polycephalum* and developed a semi-in vitro assay for DNase, wherein DNase activity was evaluated by monitoring the changes in the length of mt-nucleoids, using isolated mitochondria from zygotes. The digestion of mt-nucleoids was specifically observed using isolated mitochondria from zygotes in the presence of Mg^2+^, suggesting that Mg^2+^-dependent DNase is involved in the digestion of mtDNA for uniparental inheritance.

## Results

### Quantification of selective digestion of uniparental mt-nucleoids during zygote maturation

**Figure 1:**
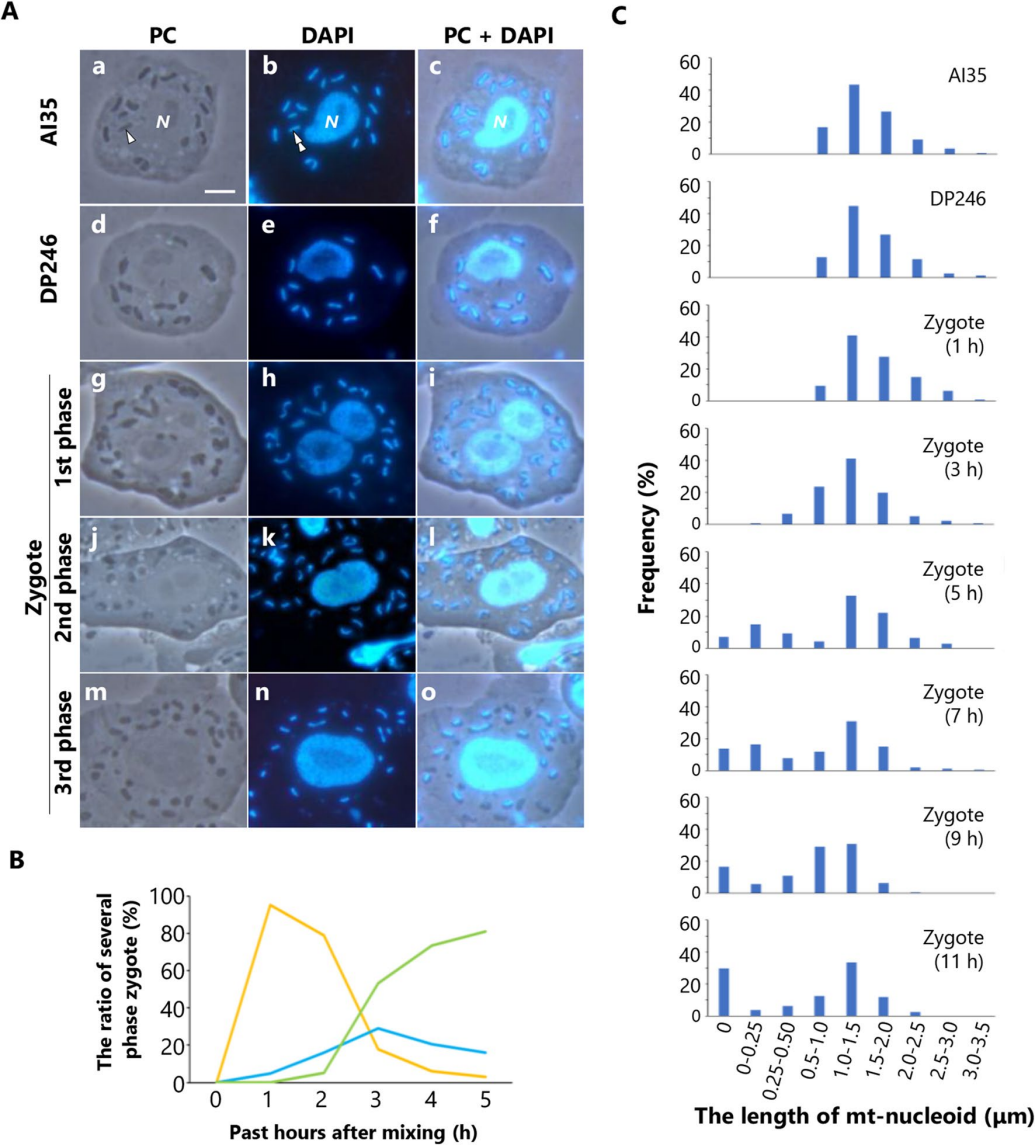
Changes in the mt-nucleoids during zygote maturation in *P. polycephalum. *(**A**) Microscopic observations of myxamoeba and zygotes in several stages. AI35 (**a**–**c**), DP246 (**d**–**f**), zygotes in first phase (**g**–**i**), zygotes in second phase (**j**–**l**), and zygotes in third phase (**m**–**o**) were fixed and stained with DAPI. Phase contrast (**a**, **d**, **g**, **j,** and **m**), DAPI (**c**, **f**, **i**, **l,** and **o**), and merged phase contrast and DAPI images are presented. Arrowhead indicates the mitochondria and double arrowhead indicates the mt-nucleoid. In zygotes in the third phase, several mitochondria had the short length mt-nucleoid. N: cell nucleus. Scale Bar = 5 μm. (**B**) Time course of the ratio of the zygotes in each phase. In the first phase (orange), each zygote had two nuclei. In the second phase (blue), two nuclei were fused, and each zygote had a nucleus with two nucleoli. In the third phase (green), two nucleoli were fused and each zygote had a nucleus with a nucleolus. Time (h) represents hours after plating. 1 h: n = 269, 2 h: n = 213, 3 h: n = 180, 4 h: n = 184, and 5 h: n = 168. (**C**) Change in the length of mt-nucleoids during zygote maturation. The length of mt-nucleoids in AI35, DP246, and zygotes at 1 h, 3 h, 5 h, 7 h, 9 h, and 11 h after plating were measured. A histogram showed that the ratio of mitochondria with mt-nucleoids of each range of length. AI35: n = 143, DP246: n = 156, zygote (1 h): n = 127, zygote (3 h): n = 136, zygote (5 h): n = 140, zygote (7 h): n = 152, zygote (9 h): n = 175, and zygote (11 h): n = 161.

In this study, two myxamoebae strains of different mating types, AI35 and DP246, were used as crosses. Both strains have no mitochondrial fusion plasmids, so mitochondrial fusion doesn’t occur. When AI35 and DP246 were crossed, previous studies showed that mt-nucleoids in mitochondria derived from DP246 were selectively digested in zygotes^23^ and mtDNA derived from AI35 was selectively inherited to plasmodium^15^. AI35 and DP246 contained 15.5 ± 1.2 and 15.7 ± 1.4 mitochondria per cell, respectively (Supplementary Fig. 1). Each mitochondrion had a long rod-shaped mt-nucleoid, and no differences in the morphology of mitochondria and mt-nucleoid were observed between AI35 and DP246 (Fig. 1A a–f). When AI35 and DP246 were mixed and plated for mating, approximately 75% of the myxamoebae synchronously fused to form zygotes. Zygote maturation proceeded in three phases. In the first phase, zygotes with two nuclei appeared, and the number of mitochondria increased to 27.6 ± 2.5 by cell fusion (Fig. 1A g–i, Supplementary Fig. 1). An hour after plating, 95% of zygotes were in the first phase (Fig. 1B). In the second phase, two nuclei were fused and zygotes became uninucleate cells with two nucleoli (Fig. 1A j–l). There were only 5% of zygotes in the second phase 1 h after plating, but their number increased 2–3 h after plating. In the third phase, two nucleoli were fused, and each nucleus had a nucleolus (Fig. 1A m–o). Zygotes in the third phase appeared 2 h after plating, and the ratio of zygotes increased to 80% 5 h after plating (Fig. 1B). As reported previously^15^, mt-nucleoids in several mitochondria became short and disappeared in zygotes 5 h after plating (Fig. 1A m–o).

We then investigated the precise timing of the digestion of the mt-nucleoids. As the length of the mt-nucleoid is proportional to the amount of mtDNA in *P. polycephalum*^19^, we measured the length of mt-nucleoids to monitor the change in the amount of mtDNA during zygote maturation. In myxamoeba, the mt-nucleoids of length 1.0–1.5 μm were most abundant, while those < 0.5 μm were not observed (Fig. 1C). The distribution was unchanged in zygotes 1 h after plating (Fig. 1). The digestion of mt-nucleoids was observed in zygotes 3 h after plating. At 3 h after plating, mt-nucleoids ≥ 0.5 μm decreased, while those < 0.5 μm appeared (Fig. 1). The ratio of mitochondria with mt-nucleoids < 0.5 μm rapidly increased from 7 to 30% in zygotes 3–5 h after plating, and then reached 38% in zygotes 11 h after plating (Fig. 1C). In addition, the ratio of mitochondria without mt-nucleoids gradually increased in zygotes 5–11 h after plating (Fig. 1C). These results indicated that the digestion of mt-nucleoids began 3 h after plating and DNase activity was maintained in the mitochondria of zygotes until at least 11 h after plating.

### Monitoring the membrane potential of mitochondria in zygote by TMRE staining

**Figure 2:**
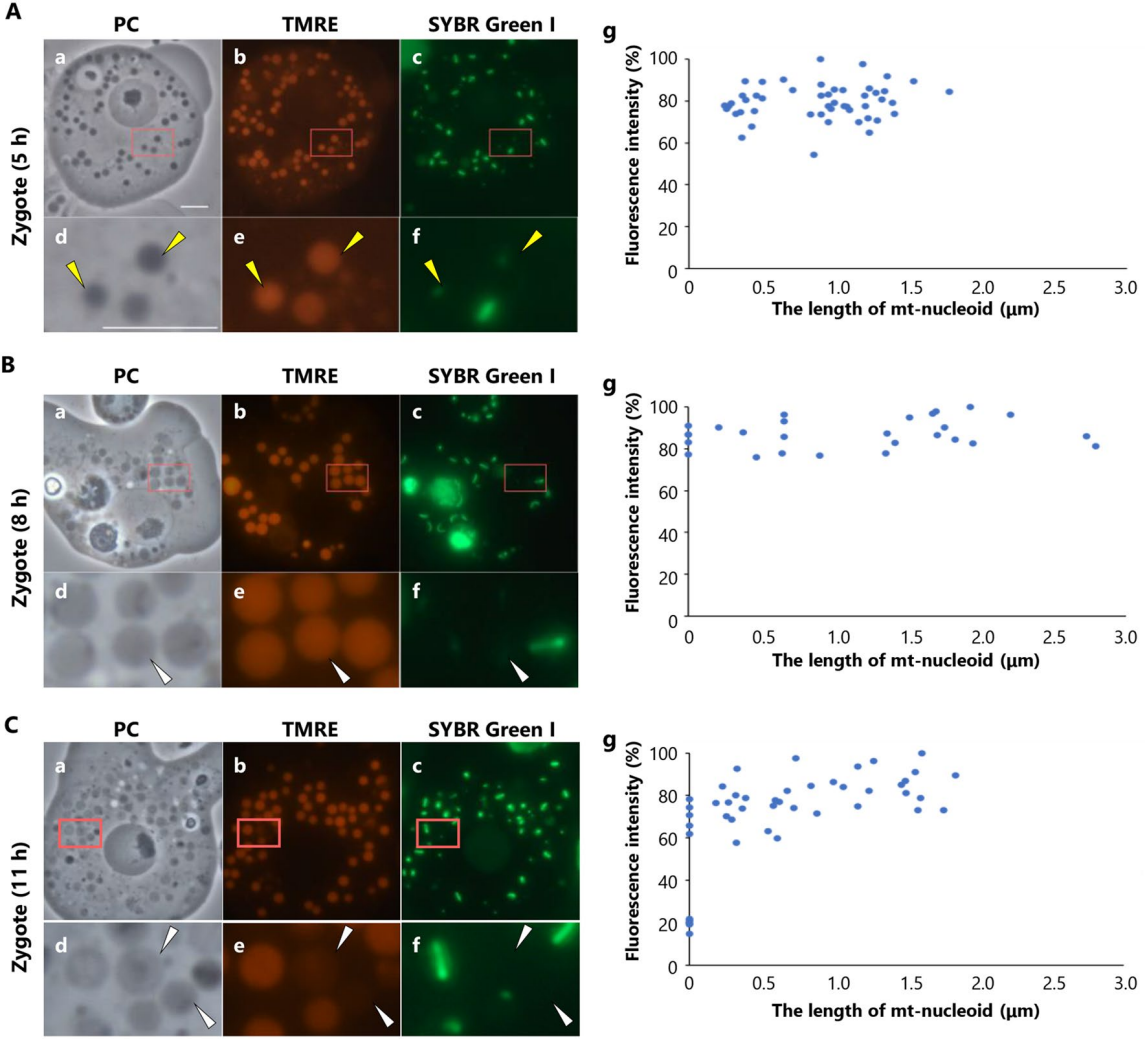
Monitoring mitochondrial membrane potential during zygote maturation using TMRE staining. (**A–C**) Observations of mitochondria stained with TMRE in zygotes at 5 h, 8 h, and 11 h after plating. Both DP246 and AI35 were stained with TMRE and then crossed. Before observations, mt-nucleoids were visualized by staining with SYBR Green I (**c**, **f**). The area enclosed by the square in each image is magnified and shown in panels (**d**–**f**). In zygotes, (**A**) at 5 h after plating, mitochondria with mt-nucleoids < 0.5 μm (yellow arrow) were observed, and (**B**, **C**) mitochondria without mt-nucleoid (white arrow) were also observed in zygotes at 8 h and 11 h after plating. In zygotes, at 11 h after plating, several mitochondria without mt-nucleoids decreased the fluorescence of TMRE (white arrow). Phase contrast (**a**, **d**), TMRE (**b**, **e**), and SYBR Green I images (**c**, **f**) are presented. Scale Bar = 5 μm. (**g**) Scatter plot of fluorescence intensity vs. the length of mt-nucleoids in the mitochondria of the zygote during digestion of mt-nucleoids. The fluorescence intensity of mitochondria stained with TMRE was plotted against the length of their mt-nucleoids in zygotes at (**A**) 5 h, (**B**) 8 h, and (**C**) 11 h after plating. The brightest signal of the TMRE in each cell was set to 100%. (**A**) n = 51, (**B**) n = 29, and (**C**) n = 48.

To check the integrity of the mitochondrial membrane during the digestion of mt-nucleoids, we monitored the mitochondrial membrane potential using tetramethylrhodamine ethyl ester (TMRE). It is known that loss of mitochondrial membrane potential by collapse of the inner membrane causes a decrease in TMRE signals^18^. Both DP246 and AI35 were stained with TMRE for 30 min and then plated for mating. After staining with SYBR Green I for visualization of mt-nucleoids, we observed mitochondria and mt-nucleoids in zygotes at 5 h, 8 h, and 11 h after plating (Fig. 2A–C). When the brightest fluorescence intensities of TMRE in each cell were set to 100%, all mitochondria, including mitochondria with mt-nucleoids < 0.5 μm had 55–100% fluorescence intensities in zygotes 5 h after plating (Fig. 2A). Since the fluorescence intensities of TMRE were not correlated with the length of mt-nucleoids in the mitochondria, the integrity of the mitochondrial membrane was considered to be maintained during the digestion of mt-nucleoids. We observed some mitochondria without mt-nucleoids in zygotes at 8 h and 11 h after plating. In zygotes 8 h after plating, mitochondria without mt-nucleoids had the same fluorescence intensity as other mitochondria with mt-nucleoids (Fig. 2B). Thus, the integrity of the mitochondrial membrane was maintained after the loss of mt-nucleoids. However, in zygotes 11 h after plating, several mitochondria without mt-nucleoids had only 20% fluorescence intensity of TMRE (Fig. 2C). This suggested that the loss of mt-nucleoids might induce the loss of mitochondrial membrane integrity.

### Characterization of DNase activity in the isolated mitochondria

To characterize the DNase involved in uniparental inheritance, we investigated whether the isolated mitochondria possessed DNase activity. We improved the methods for isolation of mitochondria from myxamoeba and zygotes and measured the length of mt-nucleoids in isolated mitochondria from cells at 0 h, 1 h, 2 h, 3 h, and 5 h after plating (Supplementary Fig. 2). Isolated mitochondria from cells at 1 h and 2 h after plating showed few mt-nucleoids of length < 0.5 μm. Similar to the in vivo results, the mt-nucleoids < 0.5 μm began to increase in the mitochondria isolated from cells 3 h after plating.

**Figure 3:**
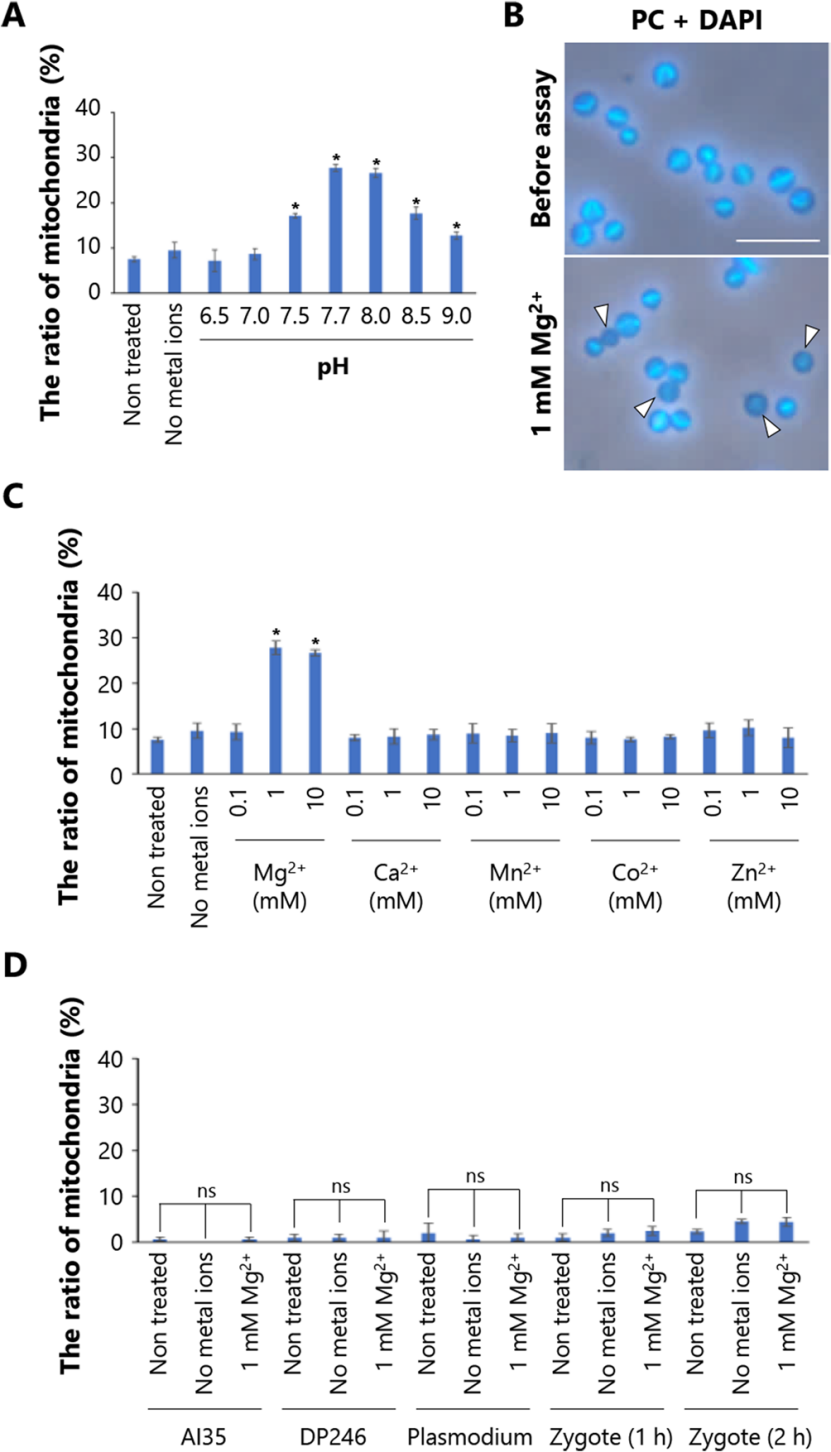
Characterization of DNase in isolated mitochondria using a semi-in vitro assay. (**A**) Effect of pH on the digestion of mt-nucleoids. The isolated mitochondria from zygotes at 3 h after plating were incubated under various pH levels (6.5–9.0) without metal ions or in the presence of five divalent ions: Mg^2+^, Ca^2+^, Mn^2+^, Zn^2+^, and Co^2+^ and then the lengths of the mt-nucleoid were measured. The ratios of mitochondria with mt-nucleoids < 0.5 μm before (non treated) or after incubation were indicated; n = 100–104 mitochondria. Error bars represent mean ± SD from three independent experiments. Significance difference was tested by Dunnett's test. *P < 0.05. (**B**) Observation of change in the length of mt-nucleoids in isolated mitochondria during incubation. The isolated mitochondria from zygotes at 3 h after plating were incubated in the presence of 1 mM Mg^2+^ at pH 7.7. The mt-nucleoids in the isolated mitochondria were observed (**a**) before, and (**b**) after incubation with DAPI staining. Arrowheads indicated the digested mt-nucleoids. Scale Bar = 5 μm. (**C**) Effect of metal ions on the digestion of mt-nucleoids. The isolated mitochondria from zygotes at 3 h after plating were incubated without metal ions or in the presence of various concentrations of Mg^2+^, Ca^2+^, Mn^2+^, Co^2+^, or Zn^2+^ at pH 7.7, and then the lengths of the mt-nucleoid were measured. The ratios of mitochondria with mt-nucleoids < 0.5 μm before or after incubation were indicated; n = 98–102 mitochondria. Error bars represent mean ± SD from three independent experiments. Significance difference was tested by Dunnett's test. *P < 0.05. (**D**) The semi-in vitro assay using the isolated mitochondria from AI35, DP246, plasmodium, and cells at 1 h and 2 h after plating. The isolated mitochondria from each cell were incubated without metal ions or in the presence of 1 mM Mg^2+^ at pH 7.7, and then the lengths of the mt-nucleoid were measured. The ratios of mitochondria with mt-nucleoids < 0.5 μm before or after incubation were indicated; n = 97–111 mitochondria. Error bars represent mean ± SD from three independent experiments. Significance difference was tested by Dunnett's test. ns: not significant.

In this study, the DNase activities were evaluated by monitoring the increase of mitochondria with mt-nucleoids < 0.5 μm in the isolated mitochondria after incubation in various conditions by a method called the semi-in vitro assay. Since the selective digestion of mt-nucleoids began to be observed in zygotes 3 h after plating, the DNase involved in uniparental inheritance is considered to function in the mitochondria of zygotes from 3 h after plating. We then performed a semi-in vitro assay using mitochondria isolated from cells 3 h after plating (Fig. 3A). We first investigated the effects of pH on the digestion of mt-nucleoids in isolated mitochondria. It is known that divalent metal ions are necessary for DNase activity, and the types of metal ions are dependent on the enzyme. Therefore, we performed this assay under various pH conditions in the presence of five divalent ions: Mg^2+^, Ca^2+^, Mn^2+^, Zn^2+^, and Co^2+^, at a concentration of 1 mM. Before the assay, 8% of mitochondria had mt-nucleoids < 0.5 μm. After incubation, the ratio of the mitochondria with mt-nucleoids < 0.5 μm increased at pH 7.5–9.0 and the highest ratio was 27% at pH 7.7, although these increases did not occur without metal ions (Fig. 3A).

Next, to identify the divalent metal ions required for DNase activity, we performed a semi-in vitro assay in the presence of various divalent metal ions at pH 7.7. When the mitochondria isolated from the cells 3 h after plating were incubated in the presence of 1 mM Mg^2+^, digestion of mt-nucleoids in mitochondria was observed (Fig. 3B). Under these conditions, the ratio of mitochondria with mt-nucleoids < 0.5 µm increased from 8 to 28% after 1 h of incubation, and also increased in the presence of 10 mM Mg^2+^ (Fig. 3B, C). However, no increase in the ratio of mitochondria with mt-nucleoids < 0.5 μm was observed in the presence of several concentrations of Ca^2+^, Mn^2+^, Co^2+^, and Zn^2+^, indicating that Mg^2+^ was necessary for DNase activity in the mitochondria isolated from cells 3 h after plating (Fig. 3C). Therefore, subsequent semi-in vitro assays were performed in the presence of 1 mM Mg^2+^ at pH 7.7.

We investigated whether Mg^2+^-dependent DNase activity was detected in mitochondria isolated from myxamoebae and plasmodia. Mitochondria with mt-nucleoids < 0.5 μm did not increase in mitochondria isolated from AI35, DP246, and plasmodium (Fig. 3D), thus indicating that DNase activity was specifically detected in mitochondria isolated from zygotes using a semi-in vitro assay. Then, to identify when the mitochondria acquired the capacity for DNase activity during zygote maturation, we performed a semi-in vitro assay using mitochondria isolated from cells at 1 h and 2 h after plating (Fig. 3D). Before performing the assay, only 1–2% of mitochondria showed mt-nucleoids < 0.5 μm. The ratio of mitochondria with mt-nucleoids < 0.5 μm did not increase after incubation, unlike for mitochondria isolated from cells 3 h after plating (Fig. 3D). The DNase activity specifically detected in the isolated mitochondria from cells 3 h after plating suggests that the Mg^2+^-dependent DNase might be involved in the selective digestion of mt-nucleoids for uniparental inheritance.

### Characterization of endonuclease G-like of *P. polycephalum*

Endonuclease G is one of the candidates for DNase involved in the selective digestion of mtDNA for uniparental inheritance^8,18^. We identified and characterized PpEndoG-like (endonuclease G-like of *P. polycephalum*) in *P. polycephalum*, which was similar to exo/endonuclease G (Supplementary Fig. 3A). PpEndoG-like is a mitochondrial protein of 407 amino acids, with a conserved DRGH/SRGH active site motif and an asparagine residue for binding the divalent metal ion. TargetP predicted that PpEndoG-like had an N-terminal mitochondrial targeting sequence (1–97 a.a.). We expressed the recombinant protein of PpEndoG-like (rPpEndoG-like), which is a predicted His-tag full-length mature protein (98–407 a.a.), and purified it using Ni^2+^ affinity chromatography. Although the purified rPpEndoG-like was in the insoluble fraction, a small amount of soluble rPpEndoG-like was obtained after dialysis in the refolding buffer (Supplementary Fig. 3B). Next, we raised a polyclonal antibody against refolded rPpEndoG-like. Western blotting analysis showed that PpEndoG-like was localized in equivalent quantities in the mitochondria of myxamoebae, zygotes, and plasmodium (Supplementary Fig. 3C). To characterize the DNase activity of PpEndoG-like, the refolded rPpEndoG-like was incubated with DNA under several conditions for 1 h, and DNA digestion was detected by agarose gel electrophoresis. The digestion of DNA was observed in the presence of Mg^2+^, Mn^2+^, and Co^2+^ at pH 5.5–8.0, and Ca^2+^ at pH 6.0–7.0 (Supplementary Fig. 3D). These metal ion requirements and the effective pH scale range were different from those for the DNase activity detected in mitochondria isolated from cells 3 h after plating.

## Discussion

Maternal/uniparental inheritance of mitochondria is a universal phenomenon in sexually reproductive species. In oogamous species, reductions in mitochondria and mtDNA occur during spermatogenesis. In contrast, in isogamous species, mtDNA in uniparental mitochondria is selectively digested during zygote maturation^15,16^. In this study, we used *P. polycephalum* to investigate the precise timing of the digestion of mt-nucleoids by monitoring the change in the length of mt-nucleoids during zygote maturation (Fig. 1A, B). The mitochondria with mt-nucleoid < 0.5 µm rapidly increased in zygotes 3–5 h after plating and mitochondria without mt-nucleoids gradually increased in zygotes 5–11 h after plating (Fig. 1B). This indicated that the digestion of mt-nucleoids was initiated in the zygotes 3 h after plating. In addition, we monitored the membrane potential of the mitochondria during zygote maturation of *P. polycephalum*. In *C. neoformans*, when a- and α-cells are crossed, mtDNA derived from the α-cell (α-mtDNA) is eliminated and only a-mtDNA is inherited. The membrane potential of α-mitochondria is maintained in dumbbell-shaped early zygotes, which contain both α-mtDNA and a-mtDNA. The elimination of α-mtDNA occurs before the digestion of α-mitochondria in vacuoles. Although the loss of membrane potential of α-mitochondria is observed in vacuoles, the exact timing of when the membrane potential decreases in *C. neoformans* is unknown*.* On the other hand, in *C. elegans*, the loss of mitochondrial membrane potential and the collapse of inner membrane in paternal mitochondria occurs rapidly after fertilization. Such collapse of the inner membrane induces translocating endonuclease G, CPS-6, from the intramembrane to the matrix, and then the paternal mitochondria and their mtDNA are selectively eliminated in embryos by mitophagy^18^. In *P. polycephalum*, we showed that the membrane potential of mitochondria was maintained during the digestion of mt-nucleoids in zygotes 5 h after plating, indicating that the integrity of the mitochondrial membrane is maintained during the digestion of mt-nucleoids (Fig. 2A). Furthermore, a decrease in the membrane potential of mitochondria without mt-nucleoids was not observed in zygotes 8 h after plating but was observed 11 h after plating (Fig. 2B, C). Rho0 cells lacking mtDNA severely reduce the mitochondrial membrane potential^24^. Therefore, the loss of mtDNA in uniparental mitochondria may induce a reduction in membrane potential. It is known that mitophagy is triggered by the loss of mitochondrial membrane potential. In mice, depolarized paternal mitochondria are degraded by mitophagy via the PARKIN and MUL1 pathways^11^. In *P. polycephalum*, the elimination of mitochondria lacking mtDNA is accomplished until 60 h after mating^15^. Thus, it is possible that the decrease in membrane potential may also be related to the elimination of mitochondria via mitophagy. Although the pathway of autophagy, including mitophagy, in *P. polycephalum*, is unknown, BLAST analysis showed that *P. polycephalum* might have some genes that are similar to ATG genes. We investigated whether mitophagy is involved in the elimination of mitochondria during uniparental inheritance in *P. polycephalum*.

Highly purified mitochondria have been isolated from *P. polycephalum* plasmodia using Percoll gradient density centrifugation^21^. These isolated mitochondria contain structurally intact mt-nucleoids and retain a high capacity for DNA synthesis. In this study, we isolated mitochondria from myxamoeba and zygotes using an improved isolation method. As expected, the ratio of mitochondria to mt-nucleoids < 0.5 µm in isolated mitochondria from cells 3 h and 5 h after plating were 8% and 36%, respectively (Supplementary Fig. 2), which is similar to those observed in vivo. We also developed a semi-in vitro assay for DNase using isolated mitochondria. In the semi-in vitro assay, DNase activity was investigated by observing the lengths of mt-nucleoids in isolated mitochondria. Moriyama et al*.* have reported the characterization of DNase activity in isolated mitochondria using DNA zymography with Native-PAGE or SDS-PAGE^25^. In DNA zymography, proteins including DNases are separated by electrophoresis, and DNase activities are visualized by staining the DNA-containing acrylamide gel with ethidium bromide. They showed that Ca^2+^-dependent DNase activity is detected in the mitochondria isolated from myxamoeba and zygotes, while Mn^2+^-dependent DNase activity is detected in mitochondria isolated from zygotes 8 h and 12 h after plating^25^. Compared to DNA zymography, the conditions of proteins and mtDNA in semi-in vitro assays are assumed to be closer to those in vivo. When mitochondria isolated from cells 3 h after plating were used in the semi-in vitro assay, the ratio of mitochondria with mt-nucleoids < 0.5 µm increased only in the presence of Mg^2+^ after incubation for 1 h but not in the presence of Mn^2+^ or Ca^2+^ (Fig. 3C). The maximum activity was obtained in the presence of 1 mM Mg^2+^ at pH 7.7 (Fig. 3C). However, Mg^2+^-dependent DNase activity was not detected in mitochondria isolated from myxamoeba, plasmodium, and zygotes 1 h and 2 h after plating (Fig. 3D). Such specific DNase activity detected only in zygotes 3 h after plating suggested that the semi-in vitro assay might be useful for characterization of DNase associated with uniparental inheritance. Thus, Mg^2+^-dependent DNase was determined to be potentially involved in the digestion of uniparental mtDNA observed in zygotes 3 h after plating.

EndoG has been reported to be involved in uniparental inheritance in *D. melanogaster*^8^ and *C. elegans*^18^. In *D. melanogaster*, paternal mtDNA is completely eliminated during spermatogenesis by two processes, and EndoG is involved in the early elimination of mtDNA^8^. In *C. elegans*, CPS-6 in paternal mitochondria translocates from the mitochondrial intermembrane space to the matrix by the collapse of the inner membrane of mitochondria after fertilization, where it might participate in paternal mtDNA degradation^18^. In contrast, in *C. neoformans*, NUC1 is not involved in the digestion of mtDNA for uniparental mitochondria^16^. In the present study, we identified and characterized PpEndoG-like proteins. The DNase activity of Pp-EndoG-like was detected in the presence of Mg^2+^, Mn^2+^, and Co^2+^ at pH 5.5–8.0 and in the presence of Ca^2+^ at pH 6.0–7.0 (Supplementary Fig. 3D). These metal requirements and the effective pH range for DNase activity were similar to those of EndoG, EXOG, CPS-6, and NUC1^26,27^ but different from DNase activity detected using semi-in vitro assays (Fig. 3C). Moreover, as described earlier, the inner membrane was maintained during mtDNA digestion in *P. polycephalum*, suggesting that translocation of PpEndoG-like protein did not occur, unlike in *C. elegans*. These results implied that endonuclease G might not be involved in the digestion of mt-nucleoids for uniparental inheritance in *P. polycephalum*.

The mechanisms of mtDNA inheritance vary among sexually reproducing species^28^. However, little is known about the evolution of maternal/uniparental inheritance. In order to understand the evolution of maternal/uniparental inheritance, it is important to investigate the regulatory mechanism of DNase for uniparental inheritance in isogamy, which is thought to be the ancestral mode of sexual reproduction. In isogamy, the DNase involved in selective digestion of mtDNA for uniparental inheritance remained elusive. In this study, the semi-in vitro assays showed that mitochondria isolated from the zygotes 3 h after plating retained the DNase involved in uniparental inheritance (Fig. 3). Compared with other species, it is expected that the mitochondria isolated from *P. polycephalum* might contain larger amounts of DNase for digestion of the large amounts of mtDNA in mt-nucleoids. In addition, approximately 75% of myxamoebae fused to form zygotes within 1 h after plating for the mating of AI35 and DP246, and zygote maturation proceeded synchronously. This mating efficiency was much higher than that of *C. neoformans*, which was only 5–10%^16^, facilitating large amounts of isolated mitochondria to be obtained from the zygotes. Such properties are advantageous for mass spectrometry (MS) analysis to identify the DNase involved in uniparental inheritance. It is expected that multiple DNases can be identified by MS analysis of isolated mitochondria because mitochondria contain several DNases involved in repair or replication of mtDNA^29^. Our findings on metal ion requirement and pH sensitivity of DNase shown in this study would provide valuable clues for identifying the DNase involved in uniparental inheritance from among multiple DNases identified by MS analysis. In addition, semi-in vitro assays also showed that Mg^2+^-dependent DNase activity was not detected in mitochondria isolated from zygotes 1 h and 2 h after plating (Fig. 3D). Further analyses to compare the components in the mitochondria of zygotes between 2 and 3 h after plating would be useful to clarify the regulatory mechanisms of selective DNase activation in uniparental inheritance.

## Methods

### Strains and culture

AI35 and DP246 were used for the myxamoebal strains of *P. polycephalum* in this study. The myxamoebae were cultured on PGY rich plates (32.3 mM KH_2_PO_4_, 11.5 mM K_2_HPO_4_, 4 mM MgSO_4_, 0.75% glucose, 1% Bacto™ Peptone, 0.1% Bacto™ yeast extract, 1.5% agarose) at 24 ℃ in the presence of live bacteria (*Aerobacter aerogenes*) as a food source.

Plasmodia of *P. polycephalum*, which is a strain of AI35 × DP246, was used. They were cultured at 21 °C in semi-defined medium as described by Daniel and Baldwin^30^.

### Zygote formation

For zygote formation, we crossed AI35 and DP246. The same amount of the two strains were mixed and then plated on SM30 mating plates (10.5 mM citric acid monohydrate, 19.5 mM trisodium citrate dihydrate, 10.0 mM MgSO_4_, 2.5% agarose). The cells were cultured at 24 °C.

### Cell preparation

The myxamoebae or zygotes grown on the plate were suspended in distilled water (DW) using a spreader and centrifuged at 1600 × g for 5 s. The cells were then washed twice with DW. Cells were suspended in DW or Isolation Buffer I (0.5 M D(-)-mannitol, 20 mM Tris–HCl (pH 7.7)) for observation of cells and isolation of mitochondria, respectively.

### Staining of mitochondria and mtDNA in live cells

Mitochondria were stained with 0.4 μg/mL TMRE (Sigma-Aldrich, St. Louis, MO, USA). To stain the mitochondria with TMRE, AI35 and DP246 were mixed and then incubated with dye at 24 °C for 30 min and then cultured on SM30 plates at 24 °C. MtDNA was stained using SYBR Green I Nucleic Acid Gel Stain (Thermo Fisher Scientific, Waltham, MA, USA), which was diluted 1:5000-times in DW.

### Staining of DNA in fixed cells

To fix the myxamoebae or zygotes, cells on the SM30 plate were fixed onto a coverslip with 8% formaldehyde and 1% glutaraldehyde in phosphate-buffered saline (PBS) at pH 7.4 for 3 min. The fixed cells were permeabilized for 5 min with 0.5% Triton-X in PBS at pH 7.4. Finally, the fixed cells were stained with 1 μg/mL 4′,6-diamidino-2-phenylindole (DAPI).

### Microscopic observation

For microscopic observations, we used a fluorescence/phase contrast microscope (BX51N-34FL; Olympus, Tokyo, Japan) connected to a 6 a pixel color camera (Axiocam 506 color; Zeiss, Oberkochen, Germany). Excitation filters, WU, WIB or WIG, were used for fluorescence observation.

### Measurement of the length of mt-nucleoids and quantification of fluorescent intensity of TMRE

Images of cells and isolated mitochondria were analyzed with ImageJ software (National Institutes of Health, http://rsb.info.nih.gov/ij/). The length of the mt-nucleoid stained with DAPI was measured using ImageJ software. The fluorescence intensity of TMRE was quantified using ImageJ.

### Isolation of mitochondria

To isolate mitochondria from the myxamoeba and zygotes**,** cells suspended in Isolation Buffer I were broken using an airbrush (Airbrush Hand-piece HP-62B, OLYMPOS, Ishikawa, Japan), which was connected to an air regulator and air compressor (APC-001; AiRTEX, Osaka, Japan), at 0.15 MPa. The broken cells were collected in a 15 mL high-clarity polypropylene conical tube with 1 mL Isolation Buffer II (0.5 M D (-)-mannitol, 20 mM Tris–HCl (pH 7.7), 1 mM EDTA (pH 7.5), 0.4 mM PMSF, 7 mM 2-Mercaptoethanol, 0.4 mM spermidine). The broken cells were centrifuged at 600 × g at 4 °C for 30 s. The supernatant was removed and centrifuged again at 9,100 × g at 4 °C for 1 min, and this supernatant was removed. The precipitated mitochondria were resuspended in Isolation Buffer II. For purification of mitochondria, the cell lysates were layered on top of a discontinuous Percoll gradient (0–40% Percoll in Isolation Buffer II). The mitochondrial fraction was collected by centrifugation at 20,400 × g at 4 °C for 45 min. The collected mitochondrial fraction was diluted in Isolation Buffer II and centrifuged at 9,100 × g at 4 °C for 1 min, after which the supernatant was removed. Mitochondria were isolated from *P. polycephalum* plasmodia, as described by Sasaki et al*.*^31^.

### Staining of DNA in isolated mitochondria

Isolated mitochondria were fixed with 0.8% glutaraldehyde in Isolation Buffer II and stained with 1 μg/mL DAPI. Before observation, fixed mitochondria were embedded in low-melting agarose type IV-A (Sigma-Aldrich) on a glass slide at 24 °C.

### Semi-in vitro assay for DNase activity

In a semi-in vitro assay for DNase activity, isolated mitochondria were incubated for 1 h at 24 °C in 1 mM MES buffer (pH 6.5) or 1 mM Tris–HCl buffer (pH 7.0–9.0), diluted using Isolation Buffer II with 0.1, 1.0, or 10 mM MgCl_2_, CaCl_2_, MnCl_2_, CoCl_2_, and ZnSO_4_, respectively. Then, the length of the mt-nucleoids stained with DAPI was measured.

## Supplementary Information

Supplementary Information.

## Data Availability

The data that support the findings of this study are available in the manuscript and its Supplementary Information files or from the corresponding author upon reasonable request.
