# Supplementary material for: Semi-in vitro detection of Mg2+-dependent DNase that specifically digest mitochondrial nucleoids in the zygote of Physarum polycephalum
Source: Sci Rep. 2022 Feb 22;12:2995. doi: 10.1038/s41598-022-06920-2 (PMC8864008; doi:10.1038/s41598-022-06920-2)
Supplement: Supplementary file 1 — Supplementary Information. [file 41598_2022_6920_MOESM1_ESM.pdf]

Supplementary Information

Semi-in vitro detection of Mg^{2+}-dependent DNase that specifically digests mitochondrial nucleoids in the zygote of *Physarum polycephalum*

Naoki Urakawa[1], Satoru Nakamura[1], Mariko Kishimoto[2], Yohsuke Moriyama[3], Shigeyuki Kawano[4], Tetsuya Higashiyama[1, 5, 6], Narie Sasaki[1, 7]

[1]Division of Biological Science, Graduate School of Science, Nagoya University, Furo-cho, Chikusa-ku, Nagoya, Aichi, 464-8602, Japan
[2] Center for the Development of New Model Organisms, National Institute for Basic Biology (NIBB) 38 Nishigonaka, Myodaiji, Okazaki, Aichi, 444-8585 Japan
[3]Science and Technology Group, Okinawa Institute of Science and Technology Graduate University (OIST), 1919-1 Tancha, Onna-son, Okinawa, 904-0495, Japan
[4]Functional Biotechnology PJ, Future Center Initiative, The University of Tokyo, 178-4-4 Wakasiba, Kashiwa, Chiba, 277-0871, Japan
[5]Institute of Transformative Bio-Molecules (WPI-ITbM), Nagoya University, Furo-cho, Chikusa-ku, Nagoya, Aichi, 464-8601, Japan
[6]Department of Biological Sciences, Graduate School of Science, The University of Tokyo, 7-3-1 Hongo, Bunkyo-ku, Tokyo, 113-0033 Japan
[7]Institute for Human Life Innovation, Ochanomizu University, 2-1-1 Otsuka, Bunkyo-ku, Tokyo, 112-8610, Japan.

Supplementary materials and methods

Expression cloning of PpEndoG-like genes: The cDNAs coding for PpEndoG-like genes were obtained by reverse transcription of mRNA isolated from AI35 using the RNeasy® Plant Mini Kit (Qiagen, Hilden, Germany). The cDNAs encoding PpEndoG-like genes without the coding regions for the respective N-terminal mitochondrial targeting sequences were amplified by PCR using KOD Neo Plus and Primer sets 1 and 2. The primer sequences used were as follows:

1. 5'- CCGAGCTCCTACCCAGCGAAGAAAATCTCCATT -3'
2. 5'- CCCAAGCTTTTACTTTTGGTCGCTTTTGGCATCTTT -3'

The cloned cDNA of the predicted mature protein of PpEndoG-like (98-407 a.a.) was inserted into the TOPO vector and transformed into *Escherichia coli* DH5α. A portion of the cDNA of PpEndoG-like was cut from the TOPO vector and inserted into the expression vector pET28a.

PpEndoG-like expression and purification: *E. coli* BL21 (DE3) bacterial cells were transformed with pET28a-PpEndoG-like. The expression of rPpEndoG-like was induced by incubation for 4 h with 1 mM IPTG at 37 °C. The bacterial cells were collected by centrifugation at 3,000 ×g at 4 °C for 15 min, and the supernatant was removed. The collected bacterial cells were suspended in sonication buffer (50 mM Tris-HCl (pH 8.0), 50 mM NaCl, 1.25 mM EDTA (pH 8.0), 250 µg/mL EDTA-free protease inhibitor cocktail) in the presence of 5 µg/µL DNase I and 0.25 mg/ml lysozyme, and then broken using an Ultrasonic Liquid Processor XL-2000 SERIES (Misonix, Farmingdale, NY, USA). The broken cells were collected by centrifugation at 9,100 ×g at 4 °C for 10 min, and the supernatant was removed. The broken cells were resuspended in sonication wash buffer (0.5 % Triton-X, 1 mM EDTA (pH 8.0), 250 µg/mL EDTA-free protease inhibitor cocktail) were sonicated and collected by centrifugation at 9,100 ×g at 4 °C for 10 min. This procedure was performed four times. The broken cells were suspended in dissolution buffer (6 M guanidinium chloride, 100 mM NaH_2PO_4, 7.6 mM Tris-HCl, pH 8.0) and shaken for 1 h at 24 ℃, and the supernatant was collected by centrifugation at 9,100 ×g at 4 °C for 30 min. The recombinant protein of PpEndoG-like was purified by Ni^{2+}-NTA-affinity chromatography using a HisTrap FF Crude column (GE Healthcare, Danderyd, Sweden).

Refolding of recombinant PpEndoG-like: The rPpEndoG-like was dialyzed into Dialysis Buffer (0.5 M NaCl, 50 mM phosphate buffer (pH 7.0)) at 4 °C overnight, and soluble rPpEndoG-like was collected as supernatant by centrifugation at 20,400 ×g at 4 °C for 1 min.

Preparation of antibody: The refolded rPpEndoG-like was used to raise polyclonal rabbit antisera. The antibodies for PpEndoG-like were purified from serum using AminoLink Coupling Resin (Thermo Fisher Scientific, Dreieich, Germany).

Western blotting: Isolated mitochondria were separated using SDS-PAGE. Proteins were transferred to polyvinylidene fluoride membranes (Immobilon®-P Transfer Membrane, 0.45 µm pore size, Merck Millipore, Darmstadt, Germany) for approximately 60 min at 100 V in a blotting buffer (2.5 mM Tris, 19.2 mM glycine, 0.02 % SDS, 20 % methanol). Membranes were blocked with 5 % non-fat milk in TBST (20 mM Tris, 150 mM NaCl, containing 0.05 % Tween-20, pH 7.4) at 4 °C overnight. Membranes were incubated with anti-PpEndoG-like or anti-ANT antibodies, which were diluted 1:500 in TBST with 1 % non-fat milk, at room temperature for 2 h. After washing the membranes with TBST with 1 % non-fat milk three times, the membranes were incubated with anti-rabbit HRP, diluted 1:30 000 in TBST with 1 % non-fat milk at room temperature for 2 h. After washing the membranes with

TBST three times and TBS (20 mM Tris, 150 mM NaCl, pH 7.4) twice, membranes were exposed to Immobilon Western chemiluminescent HRP substrate (Merck Millipore) for 1 min at room temperature and visualized using an ImageQuant LAS 4000 mini (GE Healthcare).

DNase activity assay of rPpEndoG-like: DNase activity of rPpEndoG-like was investigated after 1 h incubation of 200 ng refolded rPpEndoG-like and 200 ng DNA in 100 mM citrate buffer (pH 4.5–5.0), 100 mM MES buffer (pH 5.5–7.0), or 100 mM Tris-HCl buffer (pH 7.0–8.5) with 0.1 mM, 0.5 mM, or 1.0 mM $MgCl_2$, $CaCl_2$, $MnCl_2$, $CoCl_2$, and $ZnSO_4$, each. The samples were separated by agarose gel electrophoresis, and DNA was stained with ethidium bromide.

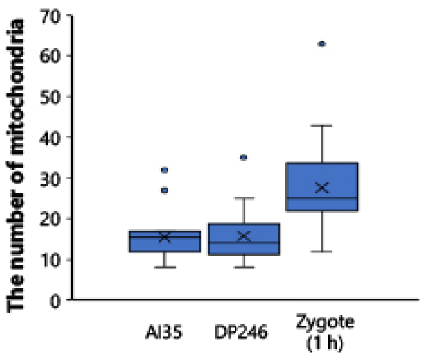

Supplementary Fig. 1 Number of mitochondria of AI35, DP246, and zygotes at 1 h after plating per cell.

Box-and-whisker plots show the number of mitochondria in AI35, DP246, and zygotes at 1 h after plating per cell. n = 20.

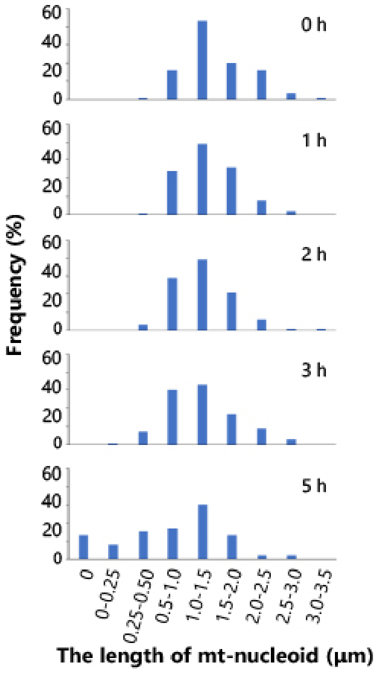

Supplementary Fig. 2 Change in the length of mt-nucleoids in isolated mitochondria from cells collected 0–5 h after plating.

Change in the length of mt-nucleoids in isolated mitochondria during zygote maturation. The length of mt-nucleoids in mitochondria isolated from zygotes at 0 h, 1 h, 2 h, 3 h, and 5 h after plating were measured. A histogram shows the ratio of mitochondria with mt-nucleoids in each range of length. A total of 100 isolated mitochondria were analyzed in each cell.

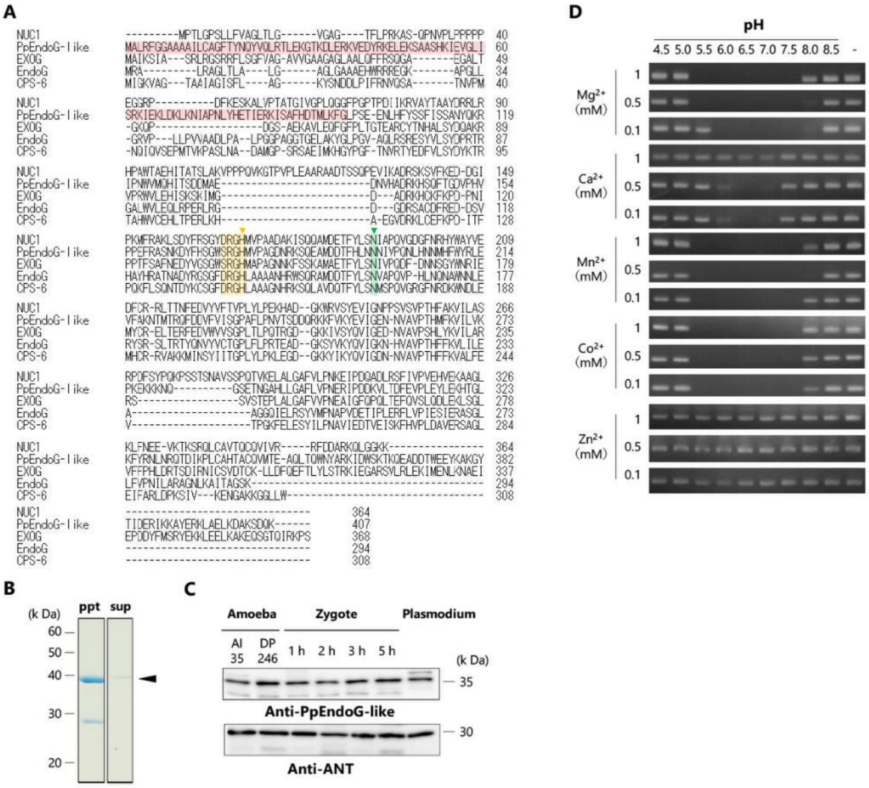

Supplementary Fig. 3 Characterization of PpEndoG-like using recombinant protein.

(**A**) Sequence alignments of PpEndoG-like, EXOG (human), NUC1 (*C. neoformans*), EndoG (human), and CPS-6 (*C. elegans*). The orange and green arrowheads mark the conserved histidine residue acting as a general base from the DRGH/SRGH active site motif and the asparagine residue binding the divalent metal ion, respectively. The red line represents the predicted region of the mitochondrial targeting sequence. (**B**) SDS-PAGE analysis of refolded rPpEndoG-like proteins. The purified rPpEndoG-like was dialyzed in refolding buffer and centrifuged. The insoluble (ppt) and soluble (sup) fractions were analyzed by SDS-PAGE. Arrowhead indicates bands of soluble rPpEndoG-like. (**C**) Western blot analysis of the expression of PpEndoG-like in isolated mitochondria from AI35, DP246, zygotes at 1 h, 2 h, 3 h, and 5 h after plating, and plasmodium. Anti-PpEndoG-like antibody or anti-ANT (adenine nucleotide translocator) antibodies were used for western blotting analysis. An anti-ANT antibody was used as a normalization control. (**D**) Characterization of the DNase activity of PpEndoG-like. The refolded rPpEndoG-like was incubated with DNA under several conditions for 1 h. After electrophoresis of each sample and untreated DNA (-), agarose gels were stained with ethidium bromide.

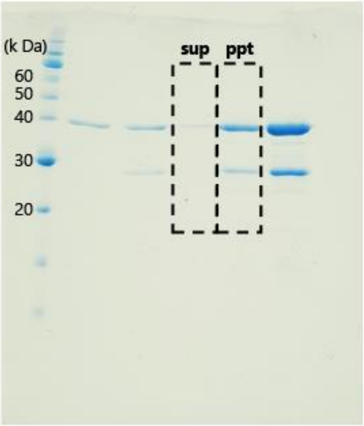

Supplementary Fig. 4 Uncropped bands of Supplementary Fig. 3B.

The protein bands shown in Supplementary Fig. 3B was indicated by boxed lines.

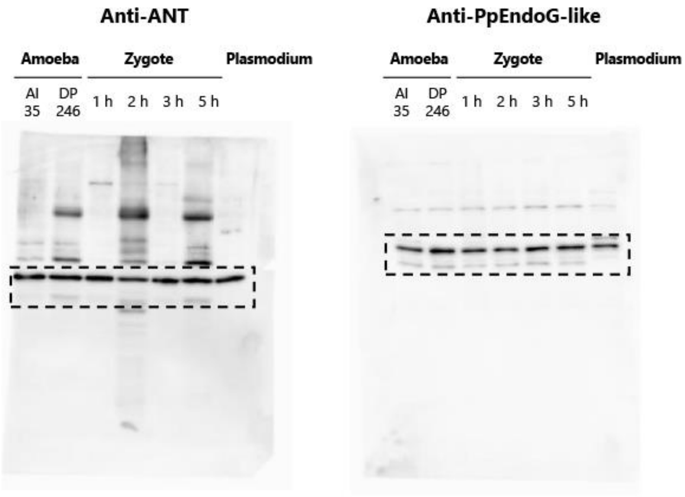

Supplementary Fig. 5 Uncropped blots of Supplementary Fig. 3C.

The protein bands shown in Supplementary Fig. 3C was indicated by boxed lines.

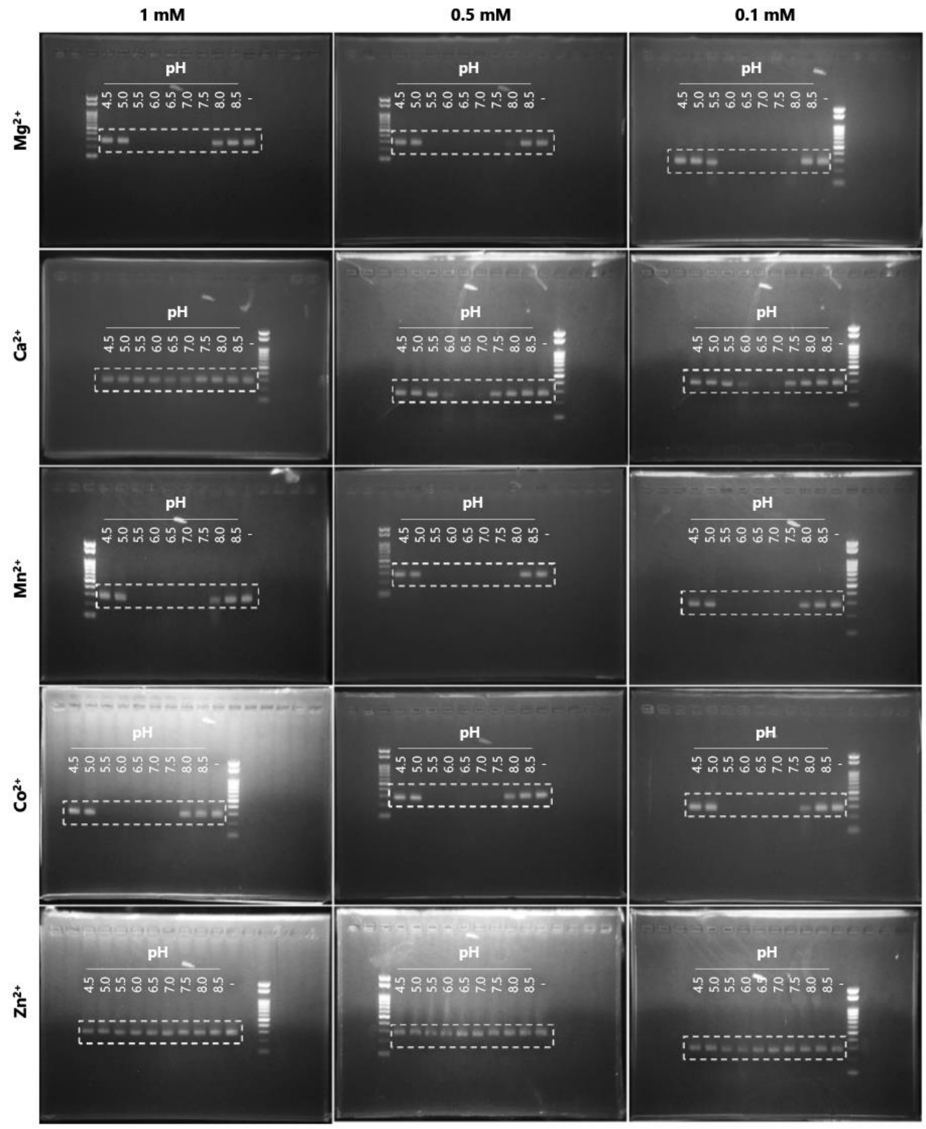

Supplementary Fig. 6 Uncropped blots of Supplementary Fig. 3D.

The DNA bands shown in Supplementary Fig. 3D was indicated by boxed lines.
